# Supplementary material for: Upper bound on the mutational burden imposed by a CRISPR-Cas9 gene-drive element
Source: G3 (Bethesda). 2026 Feb 18;16(8):jkaf315. doi: 10.1093/g3journal/jkaf315 (PMC13439934; doi:10.1093/g3journal/jkaf315)
Supplement: Supplementary file 1 [file jkaf315_supplementary_data.zip › File_S2_G3-2025-406446.pdf]

Supplemental Figures

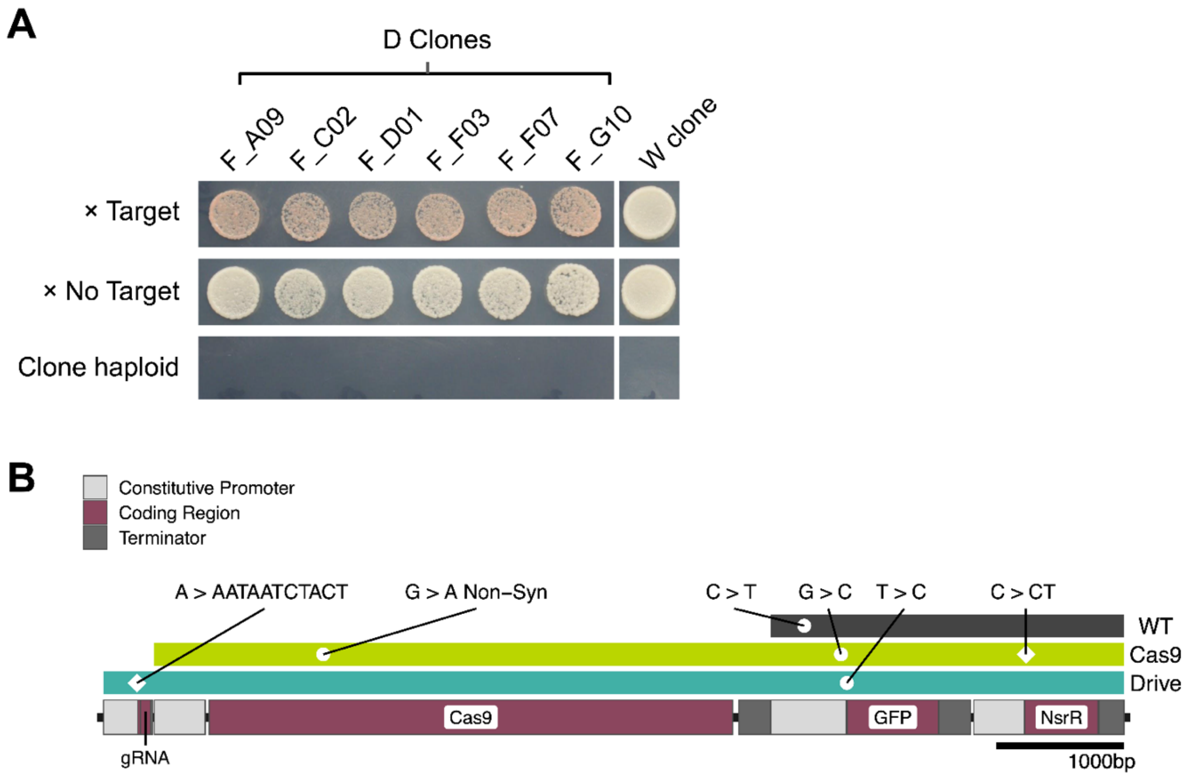

Figure S1. CCGD remains active throughout the MA experiment. A. All tested D end-point clones retain drive activity (see Section "Testing of gene-drive activity" in Materials and Methods). A haploid offspring of each tested D clone (columns) was mated either with a tester strain with the sequence targeted by the gRNA (Target) or with a tester strain without the target sequence (No target). Mated and unmated ("Clone haploid") liquid cultures were spot plated on CSM + 5FOA agar, then replica plated on CSM + G418 agar for diploid selection and incubated for 48 hours. A successful disruption of the ADE2 gene by the CCGD results in red colony coloration (DiCarlo et al. 2015). **B.** Mutations found in the construct. The construct map shows the position of each component and the three colored bars above represent the portion of the construct integrated into each strain. Circles indicate SNMs and diamonds indicate indels.

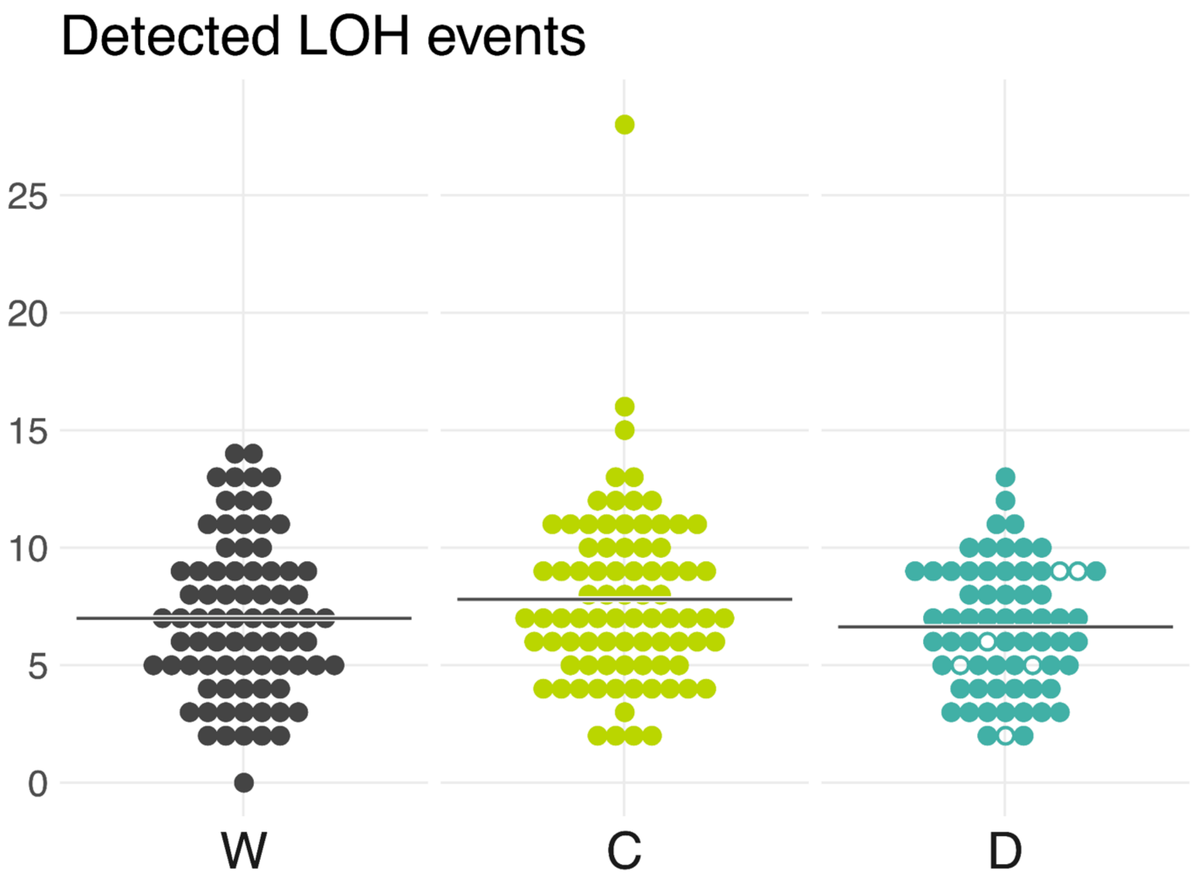

Figure S2. Distributions of detected LOH events across MA lines. Same as Figure 2B, but without corrections for undetected events.

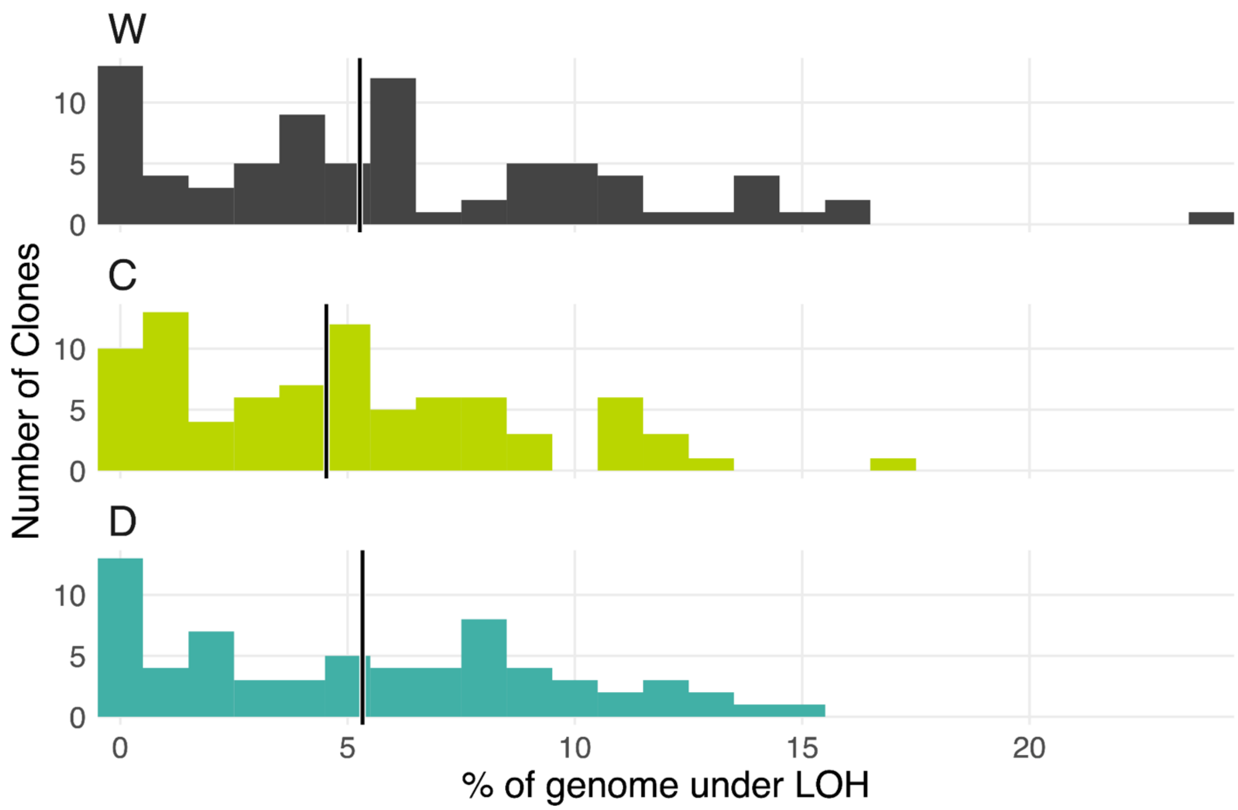

Figure S3. Distributions of LOH conversion rates among clones.

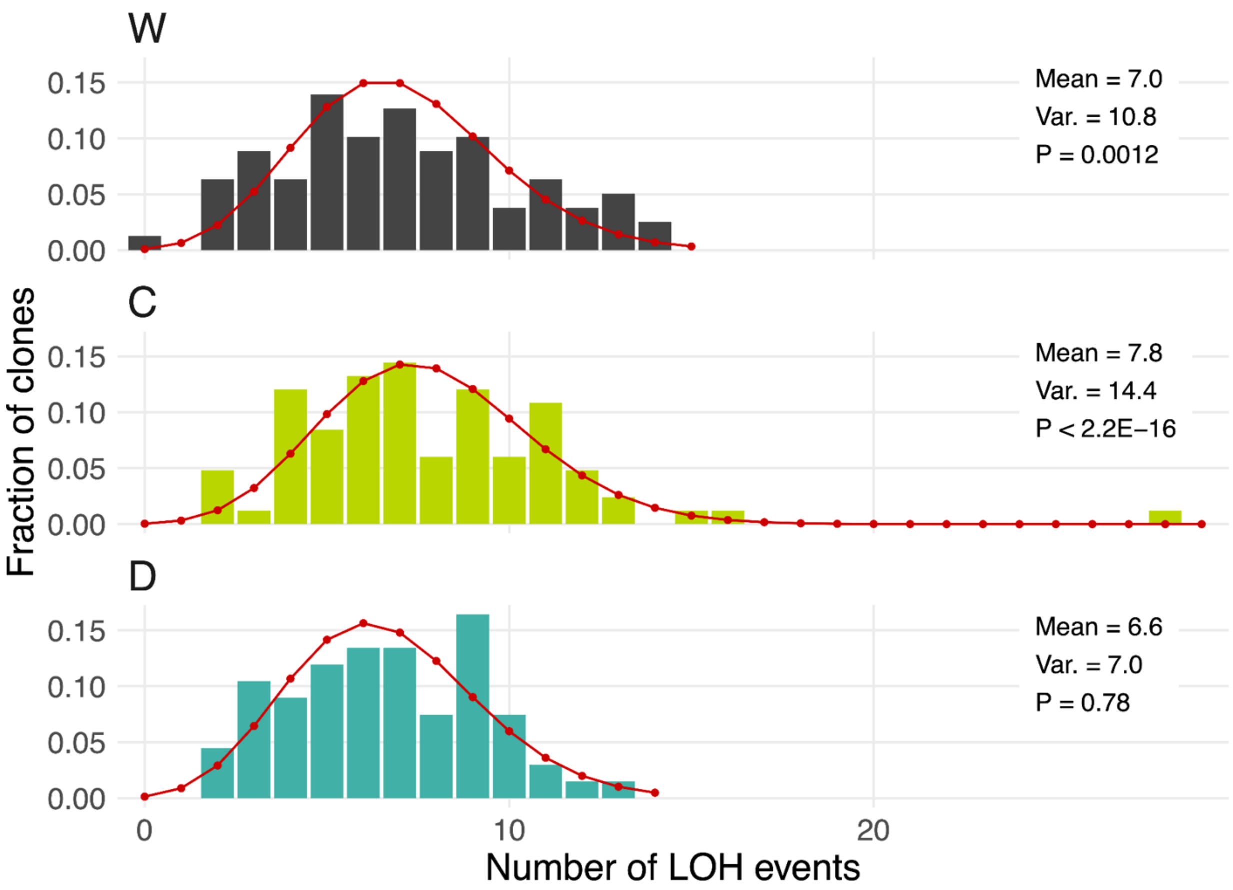

Figure S4. The distribution of LOH events across clones. The best-fit Poisson distribution is shown in red for each strain. *P*-value is calculated for the best-fit Poisson distribution based on the χ^2 goodness-of-fit test.

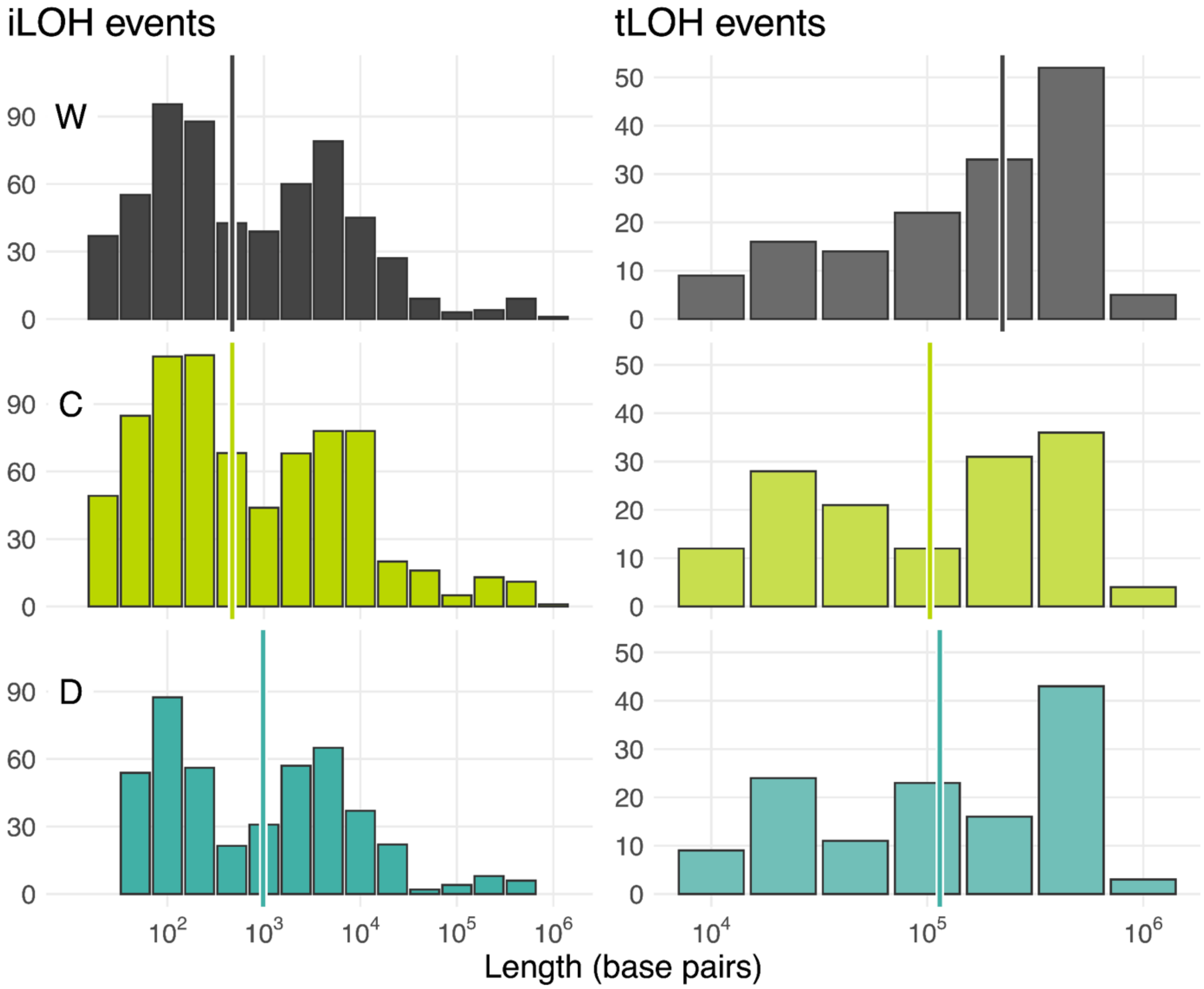

Figure S5. iLOH and tLOH length distributions across the three strains.

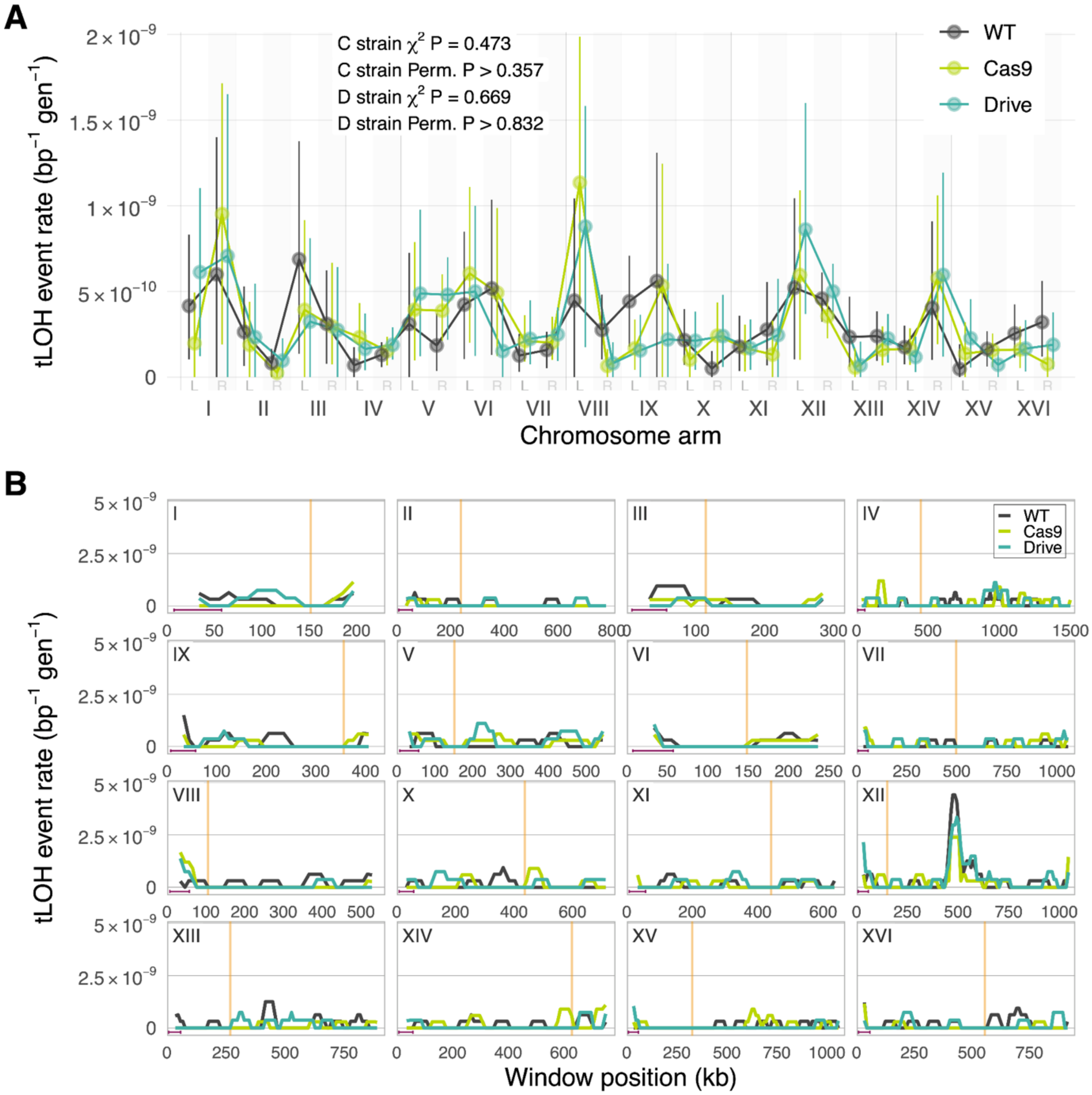

Figure S6. Distribution of tLOH event rates across the genome. Same as Figure 3, but for tLOH events.

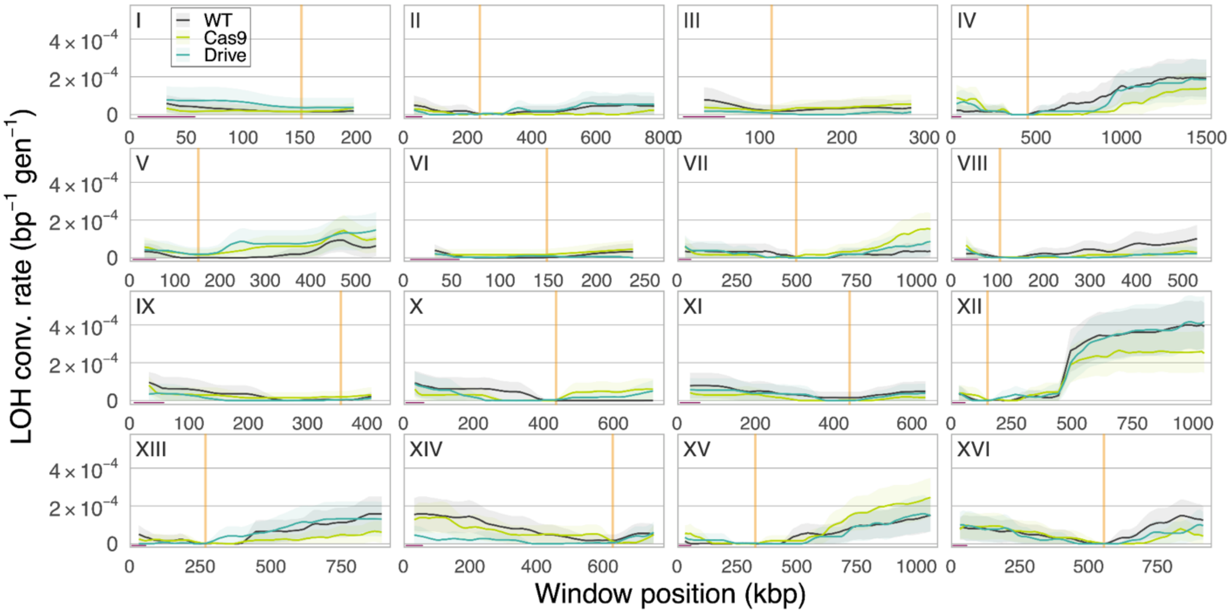

Figure S7. Distribution of LOH conversion rates across the genome. Each line shows the LOH conversion rate estimated within 50 kb sliding windows at 10 kb step sizes. Ribbons indicate 95% bootstrap confidence intervals. Maroon line segments are 50 kb scale bars, orange vertical lines are centromeres.

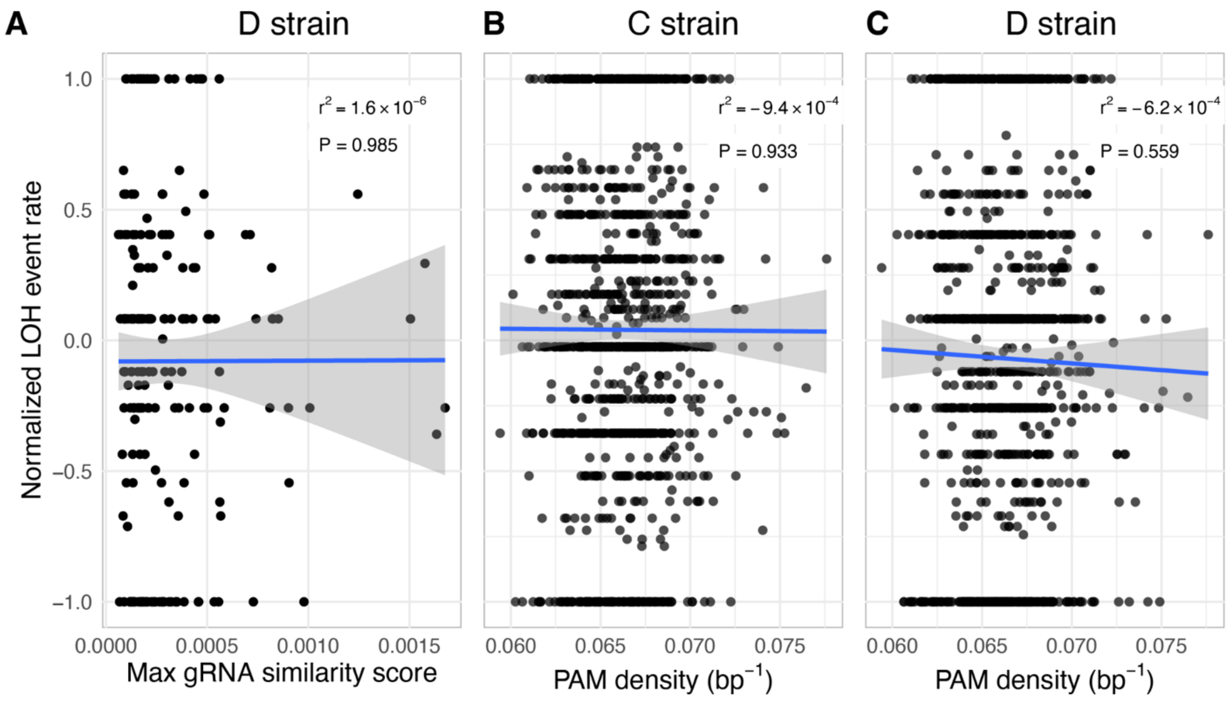

Figure S8. Correlation between the LOH event rate and sequence features across 50 kb genomic windows. Each point represents a 50 kb window in the genome. LOH event rates for C and D strains are normalized to the W rate in the same window as described in Section "Comparison of local LOH rates across strains" in the Materials and Methods. **A.** Normalized rate is plotted against the maximum similarity score between the PAM-adjacent genomic sequences and the gRNA found in a window. **B, C.** Normalized rate is plotted against the density of PAM sites in a window. For all panels, each point is a genomic window, blue lines are linear regressions, gray areas represent 95% confidence intervals.
